# Draft genome sequence of *Solimonas* sp. strain SE-A11 isolated from the rhizosphere of feral *Brassica napus* in South Korea

**DOI:** 10.1128/mra.01052-24

**Published:** 2025-02-25

**Authors:** Jihoon Kim, Kyong-Hee Nam, Jun-Woo Lee, Seong-Jun Chun

**Affiliations:** 1 LMO Team, National Institute of Ecology, Geumgang-ro, Maseo-myeon, Seocheon, South Korea; 2 Department of Biology, Wonkwang University, Iksan-daero, Iksan, South Korea; University of Guelph, Guelph, Ontario, Canada

**Keywords:** *Solimonas* sp., genome, rhizosphere, *Brassica napus*

## Abstract

The draft genome of *Solimonas* sp. strain SE-A11 bacterium isolated from the rhizosphere of feral *Brassica napus* in South Korea, represents the first isolate of the genus *Solimonas* obtained from a plant rhizosphere. The strain has a genome size of 4,937,440  bp, a G + C content of 66.5%, and 281 predicted subsystems.

## ANNOUNCEMENT

Feral *Brassica napus*, or rapeseed, thrives in natural environments, making its rhizosphere a crucial area for studying ecological interactions between plants and soil microorganisms (1, 2). *B. napus* is also a representative crop that has been widely developed and cultivated as a genetically modified (GM) crop, with instances of environmental leakage being reported (3). The isolation of *Solimonas* sp. from the lateral root of feral *B. napus* presents significant potential for its use as a bioindicator species in environmental risk assessments of the rhizosphere of genetically modified *B. napus*, establishing it as a key species for evaluating the ecological stability of rhizospheres influenced by genetically modified crops. This strain is the first *Solimonas* species (family Nevskiaceae) isolated from a plant rhizosphere, despite the genus having seven previously described species (4). Genome sequencing data can provide crucial insights into the fundamental molecular and evolutionary characteristics of this strain. Here, we report a draft genome sequence of *Solimonas* sp. strain SE-A11.

During environmental risk assessments of feral *B. napus* conducted in Naju, South Korea (35°00′3.16″N, 126°42′7.58″E) (1), bacterial strains were isolated from the lateral root using Reasoner’s 2A Agar (R2A) (Difco, MI, USA) at room temperature. The lateral roots were placed in distilled water (DW) and shaken, then serially diluted with sterilized distilled water (pH 7.0) and spread on plates. Single colonies were subcultured onto fresh R2A agar plates and incubated under identical conditions. The isolates were routinely maintained by transferring them onto new R2A plates every 10–14 days. The genomic DNA was extracted directly from plate colonies using a FastDNA SPIN Kit for Soil according to the manufacturer’s instructions (MP Biomedicals, OH, USA). The concentration and quality of extracted DNA were evaluated using a Nanodrop 2000 spectrophotometer (Thermo Scientific, DE, USA). The extracted gDNA was then used for amplification of the 16S rRNA gene and library construction. PCR was performed using the universal 16S primers 27F and 1492R (5). PCR amplification was performed under the following conditions: initial denaturation at 95°C for 5 min; 30 cycles of 95°C for 1.5 min, 55°C for 1 min, and 72°C for 1.5 min; followed by a final extension at 72°C for 10 min. Then, BLAST analysis showed 98.6% similarity to *S. fluminis* strain HR-BB (GenBank accession number NR179743.1) based on the 16S rRNA gene sequence (NCBI BLAST rRNA_typestrains/16S_ribosomal_RNA, 09.25.2024) (6). The library for whole-genome sequencing was constructed using the TruSeq Nano DNA LT Library Prep Kit (Illumina, San Diego, CA, USA), following the manufacturer’s regular protocol (insert size 350 bp), then sequenced using the HiSeq Rapid SBS Kit v2-HS with the HiSeq X platform (Illumina). After sequencing, 14,956,918 reads were produced, and total bases are 2,258,494,618. Quality control was performed using FastQC (https://www.bioinformatics.babraham.ac.uk/projects/fastqc) (7). *De novo* assembly was performed using SPAdes v3.10.1 to assemble the trimmed paired-end reads into contigs (8). Contigs shorter than 500  bp were eliminated. The CG view server constructed a circular genomic map from the resultant genome (https://cgview.ca/) (9). Annotation was performed using the RAST server (http://rast.nmpdr.org) (10). The isolate has been deposited in both the Korean Culture Center of Microorganisms (KCCM 43467) and the Japan Collection of Microorganisms (JCM 36557).

**Fig 1:**
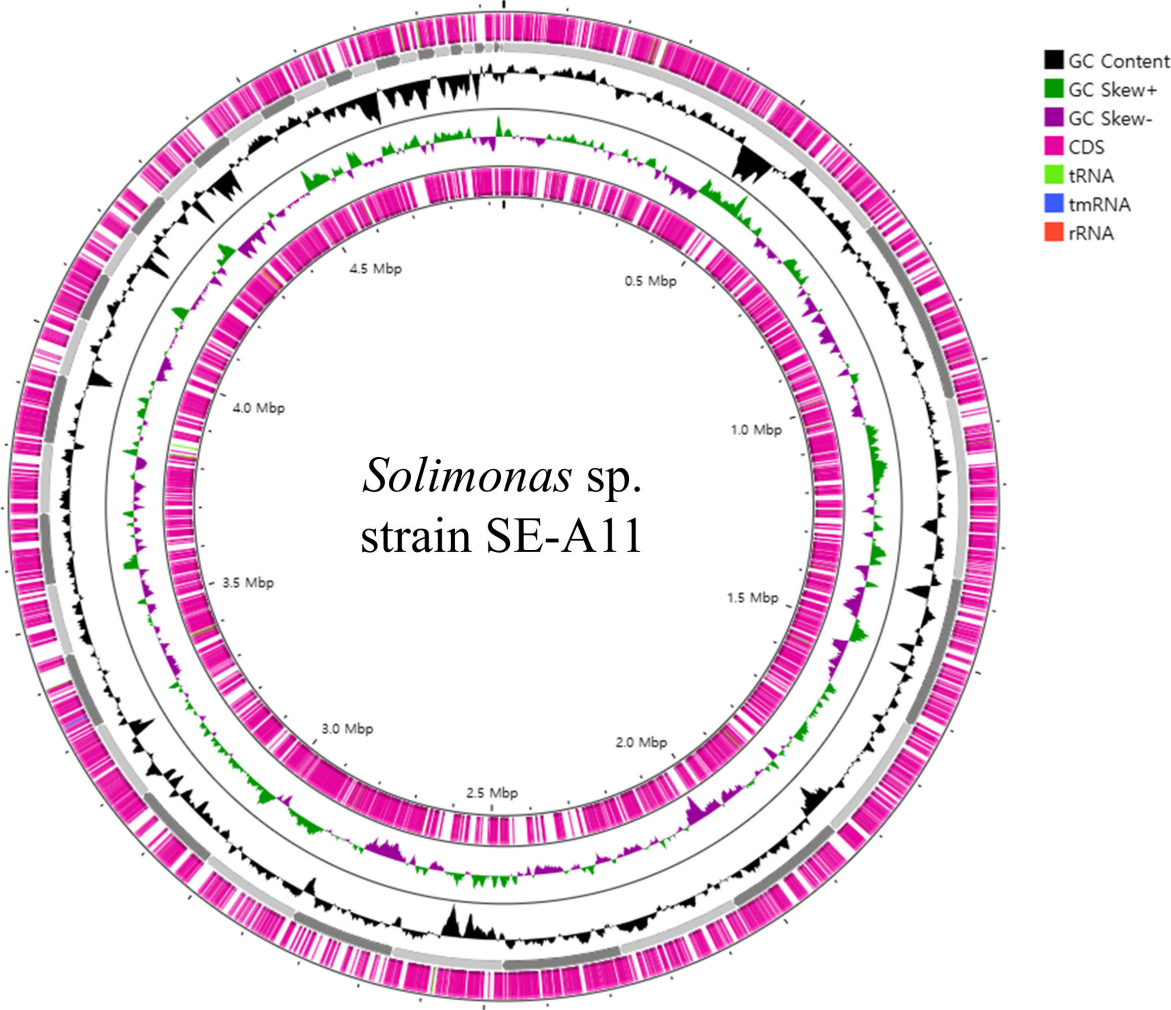
Genome map of *Solimonas* sp. strain SE-A11 built using CGView server (https://proksee.ca/ accessed on 25 September 2024). Genomic features are represented from the outer to the inner circle, circle 1—illustrates Prokka annotated forward and coding sequences (CDS) with tRNA, rRNA, and tmRNA; circle 2—represents the GC content; circle 3—displays the GC skew (G-C) / (G + C).

The genome consists of 42 contigs, totaling 4,937,440 bp, with an N50 value of 203.8 kb and a high G + C content of 66.5% (Fig. 1). The genome coverage was 149×, indicating a high level of sequencing depth and reliability in the genome assembly. A total of 4,525 protein-coding genes and 55 RNA genes were predicted, with the genes classified into 281 subsystems.

## Data Availability

The 16S rRNA gene sequence, draft genome assembly, and raw reads of Solimonas sp. strain SE-A11 have been deposited in GenBank under accession numbers ON126223 , assembly accession number NZ_JAUASX000000000.1 , BioProject accession number PRJNA983434, Assembly accession number GCF_030345715.1 , and raw reads accession number PRJNA1205544.
